# Smoking status and anti-inflammatory macrophages in bronchoalveolar lavage and induced sputum in COPD

**DOI:** 10.1186/1465-9921-12-34

**Published:** 2011-03-22

**Authors:** Lisette IZ Kunz, Thérèse S Lapperre, Jiska B Snoeck-Stroband, Simona E Budulac, Wim Timens, Simone van Wijngaarden, Jasmijn A Schrumpf, Klaus F Rabe, Dirkje S Postma, Peter J Sterk, Pieter S Hiemstra, Groningen Leiden Universities Corticosteroids in Obstructive Lung Disease (GLUCOLD) study group

**Affiliations:** 1 Department of Pulmonology, Leiden University Medical Center, Leiden, The Netherlands; 2 Department of Medical Decision Making, Leiden University Medical Center, Leiden, The Netherlands; 3 Department of Epidemiology, University Medical Center Groningen, University of Groningen, Groningen, The Netherlands; 4 Department of Pathology, University Medical Center Groningen, University of Groningen, Groningen, The Netherlands; 5 Department of Pulmonology, University Medical Center Groningen, University of Groningen, Groningen, The Netherlands; 6 Department of Pulmonology, Academic Medical Center, Amsterdam, The Netherlands

## Abstract

**Background:**

Macrophages have been implicated in the pathogenesis of COPD. M1 and M2 macrophages constitute subpopulations displaying pro- and anti-inflammatory properties. We hypothesized that smoking cessation affects macrophage heterogeneity in the lung of patients with COPD. Our aim was to study macrophage heterogeneity using the M2-marker CD163 and selected pro- and anti-inflammatory mediators in bronchoalveolar lavage (BAL) fluid and induced sputum from current smokers and ex-smokers with COPD.

**Methods:**

114 COPD patients (72 current smokers; 42 ex-smokers, median smoking cessation 3.5 years) were studied cross-sectionally and underwent sputum induction (M/F 99/15, age 62 ± 8 [mean ± SD] years, 42 (31-55) [median (range)] packyears, post-bronchodilator FEV_1 _63 ± 9% predicted, no steroids past 6 months). BAL was collected from 71 patients. CD163^+ ^macrophages were quantified in BAL and sputum cytospins. Pro- and anti-inflammatory mediators were measured in BAL and sputum supernatants.

**Results:**

Ex-smokers with COPD had a higher percentage, but lower number of CD163^+ ^macrophages in BAL than current smokers (83.5% and 68.0%, p = 0.04; 5.6 and 20.1 ×10^4^/ml, p = 0.001 respectively). The percentage CD163^+ ^M2 macrophages was higher in BAL compared to sputum (74.0% and 30.3%, p < 0.001). BAL M-CSF levels were higher in smokers than ex-smokers (571 pg/ml and 150 pg/ml, p = 0.001) and correlated with the number of CD163^+ ^BAL macrophages (Rs = 0.38, p = 0.003). No significant differences were found between smokers and ex-smokers in the levels of pro-inflammatory (IL-6 and IL-8), and anti-inflammatory (elafin, and Secretory Leukocyte Protease Inhibitor [SLPI]) mediators in BAL and sputum.

**Conclusions:**

Our data suggest that smoking cessation partially changes the macrophage polarization *in vivo *in the periphery of the lung towards an anti-inflammatory phenotype, which is not accompanied by a decrease in inflammatory parameters.

## Background

Chronic obstructive pulmonary disease (COPD) is characterized by progressive lung function decline and an abnormal inflammatory response in the airways, mainly caused by cigarette smoke [1]. The inflammation response in the small airway in COPD is characterized by the accumulation of macrophages, neutrophils, CD8^+^-lymphocytes and B-cells and is associated with the severity of COPD [2,3]. Smoking cessation is an effective treatment to reduce lung function decline [1]. Nevertheless, airway inflammation in bronchial biopsies, sputum and bronchoalveolar lavage (BAL) of COPD patients (predominantly) persists one year after smoking cessation [4-6]. We previously showed that the number of macrophages and neutrophils in bronchial biopsies are comparable in current and ex-smokers with COPD [7]. However, the effects of smoking on macrophage phenotypes in COPD are incompletely understood.

Macrophages play an important role in innate and adaptive immunity and form a heterogeneous population [8,9]. Macrophages display polarized phenotypes by which they can be divided into subpopulations. Pro-inflammatory, or classically activated macrophages (M1) display pro-inflammatory and cytotoxic properties and can eradicate intracellular pathogens. In contrast, anti-inflammatory or alternatively activated macrophages (M2) display anti-inflammatory properties and are implicated in repair [8,10]. Granulocyte-macrophage colony stimulating factor (GM-CSF) can generate M1 *in vitro *from human peripheral blood monocytes, and macrophage colony stimulating factor (M-CSF) can generate M2 [11]. M1 secrete pro-inflammatory cytokines, like IL-(Interleukin)-12 and tumor necrosis factor (TNF)-α, have good antigen presenting capacity and promote Th1 immunity. In contrast, M2 secrete anti-inflammatory mediators, such as IL-10, show poor antigen presenting capacity and promote development of T-regulatory cells [11-13]. Alveolar macrophages show anti-inflammatory M2-characteristics [14-16], which can be distinguished from pro-inflammatory macrophages using M2 markers such as the scavenger receptor CD163 [17,18]. Compared to M1 cells, M2 macrophages are highly phagocytic. The phagocytic capacity of alveolar macrophages is decreased in smoking COPD patients and improves with smoking cessation [19]. This suggests a phenotypic alteration and a role of macrophage heterogeneity in COPD, which has also been proposed in e.g. tumor progression [20], atherosclerosis [21] and renal diseases [22].

Although inflammation persists, smoking cessation shows positive clinical effects [1]. This suggests that other mechanisms play a beneficial role, for instance regulation of macrophage polarization. We hypothesize that in moderate to severe COPD patients *i*) ex-smokers have more M2 and anti-inflammatory mediators in BAL and induced sputum compared to current smokers; *ii*) M2 and anti-inflammatory mediators are relatively higher in the peripheral airways (as sampled by BAL) than in the central airways (as sampled by induced sputum).

## Methods

### Subjects and study design

Patient characteristics and methods have been described previously [7,23,24]. In short, we studied 114 clinically stable moderate to severe COPD patients [GLUCOLD study (Groningen Leiden Universities Corticosteroids in Obstructive Lung Disease)] cross-sectionally. They were aged 45-75 years, smoked ≥10 packyears and were current or ex-smokers (quit ≥1 month). Patients diagnosed with asthma, α1-antitrypsin deficiency and those who used corticosteroids in the past six months were excluded; they were allowed to use short-acting bronchodilators. Approval of the medical ethics committees of both centers was obtained and all patients provided written informed consent [23]. Spirometry was performed according to international guidelines [25]. All patients underwent a bronchoscopy with BAL and a sputum induction on separate visits.

### Bronchoscopy, BAL and sputum induction

Fiberoptic bronchoscopy was performed in all patients and processed using a standardized protocol, as previously described [7,24,26,27]. The BAL procedure was discontinued during the study due to ethical considerations, since four of 71 patients experienced a serious adverse event that was considered to be possibly related to the BAL procedure (pleural pain, fever, pneumonia, short-term cardiac ischemia). Sputum induction was achieved using hypertonic sodium chloride aerosols (w/v 4.5%) for a maximal duration of three times five minutes and processed according to the whole sample method.

### BAL and sputum processing

BAL was filtered through a nylon gauze and centrifuged for 10 minutes at 450*g at 4°C. If erythrocytes were macroscopically present, the cell pellet was resuspended in lysisbuffer (100 ml phosphate buffered saline (PBS) containing 0.83 gram NH_4_Cl, 0.1 gram KHCO_3 _and 0.004 gram Ethylenediaminetetra Acetic Acid (EDTA), pH 7.4) for 5 minutes and centrifuged (450*g, 4°C). The cell pellet was resuspended in 0.1% glucose (w/v) in PBS and centrifuged again under the same conditions. BAL processing and differential cell counts were performed analogous to the methods described for sputum processing, except that no dithiothreitol was used for homogenization. The viability of the non-squamous cells in BAL was similar in smokers and ex-smokers (82 ± 12% versus 82 ± 9%, p = 0.96).

Sputum was processed according to the whole sample method and all samples were treated with dithiothreitol 0.1% (DTT, Sputolysin, Calbiochem) [28]. Cell free supernatants of both BAL and sputum were stored at -80°C.

From both BAL and sputum samples cytospins were centrifuged on apex-coated slides [28]. A sputum sample was considered adequate when the percentage squamous cells was less than 80%. After drying for 1 hour, the cytospins were wrapped in aluminum foil and stored at -80°C pending immunocytochemical staining.

### Immunocytochemical staining

Frozen cytospins of BAL and sputum were brought to room temperature in one hour. BAL cytospins were fixed in acetone at -20°C for 10 minutes, dried and endogeneous peroxidase activity was blocked by incubation in methanol and 0.3% hydrogen peroxide for 10 minutes. Sputum cytospins were fixed in 4% paraformaldehyde in PBS 0.9% (w/v) for 1 hour, rinsed with PBS and endogenous peroxidase activity was blocked with sodium azide 0.1% (w/v) and hydrogen peroxide 0.18% (w/v) in PBS for 30 minutes. Non-specific binding was blocked in PBS, 1% bovine serum albumin (BSA) and 5% normal human serum (NHS) for 45 minutes for the sputum cytospins only. Mouse-anti-human CD163 (clone GHI/61, BD Pharmingen) was used as a primary antibody to stain M2-type macrophages [17] at the dilution of 1:75 for BAL cytospins and 1:50 for sputum cytospins, and both were incubated for one hour at room temperature. The primary antibody was diluted in PBS/1% BSA for BAL cytospins and in PBS/1%BSA/1%NHS for sputum cytospins. The horseradish peroxidase conjugated anti-mouse Envision system (DAKO, Glostrup, Denmark) was used as a secondary antibody and was incubated for 30 minutes, the chromogen NovaRed (Vector, Burlingame, CA) for 7 minutes. All washing steps were with PBS. All slides were counterstained with Mayer's hematoxylin (Klinipath, Duiven, The Netherlands) and mounted afterwards with Pertex mounting medium (HistoLab, Gothenburg, Sweden).

We considered the possibility that DTT used to liquefy the induced sputum samples affects detection of CD163. To this end we generated M1 and M2 by culture of monocytes for six days in the presence of GM-CSF and M-CSF respectively [11], and treated these cells with DTT prior to FACS-based analysis of CD163 expression and preparation of cytospins followed by immunocytochemical staining for CD163.

### Analysis of cytospins

**Figure 1:**
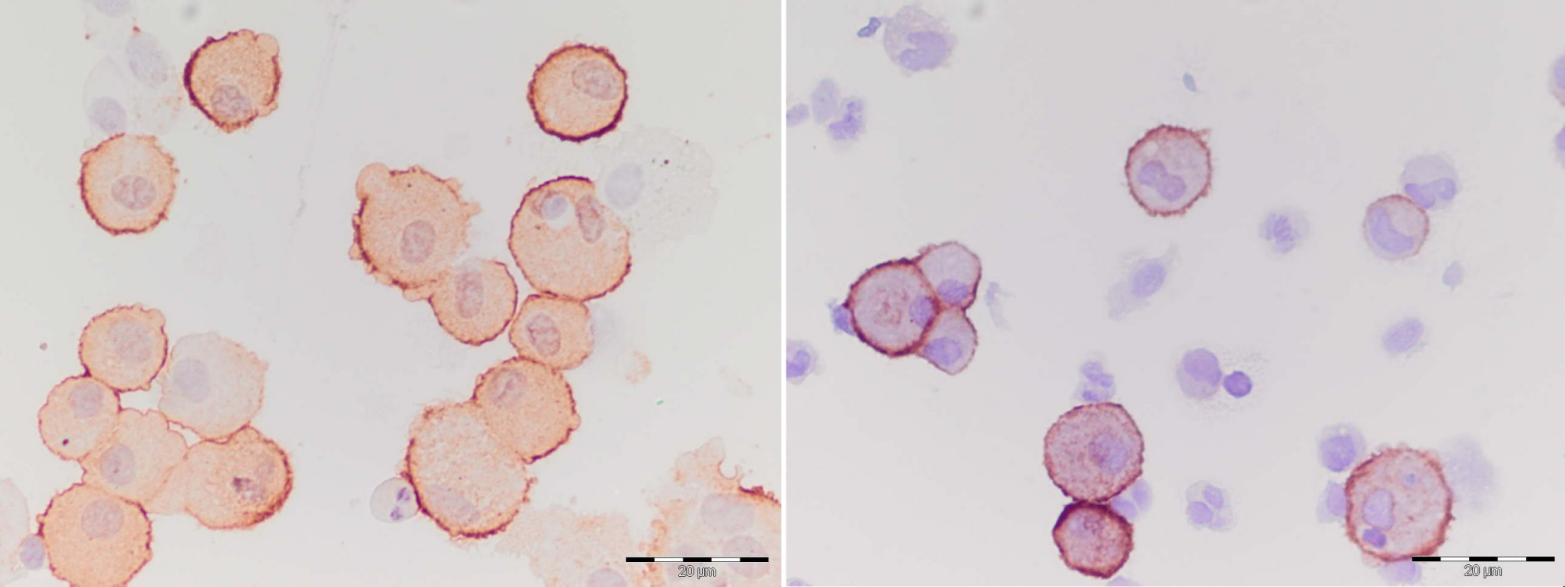
**Photomicrograph of membrane-bound CD163 staining on BAL and sputum cells**. A BAL cytospin is shown in the left photograph and a sputum cytospin in the right photograph. Scale bar = 20 μm

Two cytospins per sample were stained for differential cell counts with May-Grünwald Giemsa (MGG). Differential cell counts were expressed as a percentage of nucleated cells, squamous cells excluded. The median percentage squamous cells was 7.5% (2.1-13.3%). CD163^+ ^and CD163^- ^macrophages were enumerated based on morphology by two independent, experienced researchers at 400× magnification (figure 1). To avoid observer bias, slides were coded without knowledge of clinical data. The mean number of CD163^+ ^macrophages divided by the total counted number of macrophages was used to calculate the percentage of CD163^+ ^macrophages. The total number of CD163^+ ^macrophages per volume was calculated by the percentage of CD163^+ ^macrophages multiplied by the total number of macrophages. Repeatability between the two observers (LIK and SVW) was good, as measured by the intraclass coefficient (ICC), with the two way random model and absolute agreement. For BAL CD163^+ ^and CD163^- ^macrophages the ICC were both 95%; for sputum CD163^+ ^and CD163^- ^macrophages the ICC were 97% and 93% respectively.

### Enzyme-linked Immunosorbent Assay (ELISA)

Commercially available kits were used to detect GM-CSF (Bender Medsystems), M-CSF (R&D systems), IL-6, IL-8, IL-10 (Sanquin), IL-12 (IL-12/IL-23p40, R&D systems) and elafin (HBT) in sputum and BAL supernatants. SLPI ELISA was developed in our laboratory at the Leiden University Medical Center [29]. The absorbance was measured at 450 nm using a Microplate reader (model 680; Bio-Rad, Hercules, CA) and Microplate Manager software (version 5.2.1, Bio-Rad). The lower limits of detection for sputum were 300 pg/ml (SLPI), 2.5 ng/ml (elafin), 38 pg/ml (IL-6) and 400 pg/ml (IL-8). The lower limits of detection for BAL were 150 pg/ml (M-CSF), 0.2 ng/ml (SLPI), 5.5 pg/ml (IL-6) and 15 pg/ml (IL-8). In BAL and sputum supernatants, IL-10, IL-12, GM-CSF levels were below the lower limit of detection. Furthermore, elafin and M-CSF were undetectable in BAL and sputum supernatants respectively. In case more than 10% of the samples were below the detection limits, the value of these samples was set at the lower limit of detection (M-CSF and IL-6 in BAL).

### Statistical analysis

Mean values and standard deviations (SD) or medians with interquartile ranges (IQR) are presented. When appropriate, variables were logarithmically transformed before statistical analysis. Differences between smokers and ex-smokers were explored using χ^2^-tests, two-tailed unpaired t-tests and Mann-Whitney tests. We used the Spearman (Rs) correlation coefficient to analyze correlations. Multiple linear regression was used to correct for the recovery of BAL. The statistical analysis was performed with SPSS 16.0 software (SPSS Inc., Chicago, IL). Statistical significance was inferred at p < 0.05.

## Results

### Characteristics

**Figure 2:**
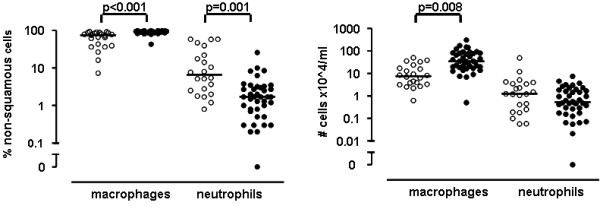
**BAL differential cell counts expressed as percentage and cell concentrations of COPD patients**. Percentage is shown in the left panel, cell concentrations in the right panel. Open circles represent ex-smokers, closed circles represent current smokers. Horizontal bars represent medians. P-values are corrected for recovery of BAL fluid using multiple linear regression.

**Figure 3:**
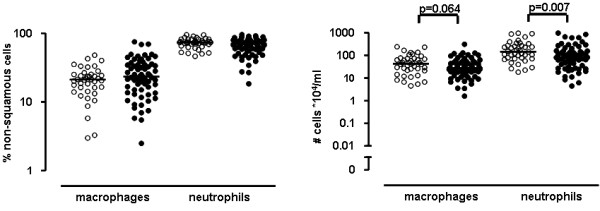
**Sputum differential cell counts expressed as percentage and cell concentrations of COPD patients**. Percentage is shown in the left panel, cell concentrations in the right panel. Open circles represent ex-smokers, closed circles represent current smokers. Horizontal bars represent medians.

In total, 114 COPD patients participated in the study, 72 current smokers and 42 ex-smokers, as presented in table 1. All steroid-naive patients had moderate to severe COPD (GOLD stage II-III) based on a mean (SD) post-bronchodilator FEV_1 _of 63 (9)% predicted and had a median (25^th ^and 75^th ^percentile) smoking history of 42 (31-55) packyears. The total group of patients and the unselected group in which BAL was performed were comparable. Of the BAL samples (first 71 patients), 62 were suitable for analysis. 106 out of 109 sputum inductions were suitable for analysis. BAL and sputum cell differentials and cell concentrations are presented in figures 2 and 3. The percentage and number of macrophages in BAL were significantly higher in current smokers than in ex-smokers (95.8% and 74.2%, p < 0.001; 34.0 and 7.6 × 10^4^/ml, p = 0.008 respectively). The mean recovery of BAL was 41 (18)%; the recovery in smokers was higher compared to ex-smokers (45 (16)% and 35 (19)%, p = 0.039 respectively).

**Table 1:** Patient characteristics for current and ex-smokers with COPD

	Smokers (n = 72)	Ex-smokers (n = 42)
Males (n (%))	59 (81.9)	40 (95.2)*
Age (years)	60.1 (7.7)	64.1 (7.2)*
Packyears	43.3 (32.4-55.6)	36.8 (27.5-53.1)
Smoking cessation (years)		3.5 (1.0-9.8)
FEV_1 _post-bronchodilator (L)	2.02 (0.46)	2.05 (0.46)
FEV_1 _post-bronchodilator (%pred)	63.3 (8.3)	62.5 (9.6)
FEV_1_/IVC% post-bronchodilator	49.5 (8.5)	46.0 (8.3)*
K_CO _(%pred)	73.3 (25.1)	80.4 (25.9)

Data are presented as mean (SD) or median (IQR) unless otherwise stated. These patient characteristics have been previously described [7].

pred = predicted; FEV_1 _= forced expiratory volume in one second; IVC = inspiratory vital capacity; K_CO _= carbon monoxide transfer coefficient.

* p < 0.05 compared with smokers with COPD (χ^2 ^test for sex differences, two tailed unpaired t-tests for other data).

### Smoking status and CD163^+ ^macrophages in BAL and induced sputum

**Figure 4:**
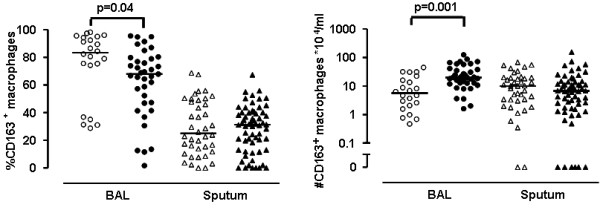
**The percentage and number of CD163^+ ^macrophages in BAL and induced sputum in COPD patients**. The percentage (left panel) and number of CD163^+ ^macrophages (right panel) in BAL and induced sputum between ex-smokers (open symbols) and smokers (closed symbols) with COPD. Horizontal bars represent medians. P-values are corrected for recovery of BAL fluid using multiple linear regression.

DTT used to liquefy the induced sputum did not affect detection of CD163 by FACS and immunocytochemical staining (data not shown). Ex-smokers with COPD had a significantly higher percentage of anti-inflammatory CD163^+ ^macrophages in BAL than current smokers (83.5% and 68.0%, p = 0.04, respectively) (figure 4), independent of BAL recovery. However, ex-smokers had a lower number of anti-inflammatory macrophages in BAL compared to current smokers (5.6 and 20.1 × 10^4^/ml, p = 0.001, respectively). The percentage CD163^+ ^macrophages was higher in BAL compared to sputum (74.0% and 30.3%, p < 0.001, respectively). Ex-smokers had a similar percentage and number of anti-inflammatory macrophages in induced sputum compared to current smokers with COPD (25.0% and 31.1%, p = 0.89; 10.1 and 6.8 ×10^4^/ml, p = 0.24 respectively).

### Smoking status and soluble mediators in BAL and induced sputum supernatants

**Figure 5:**
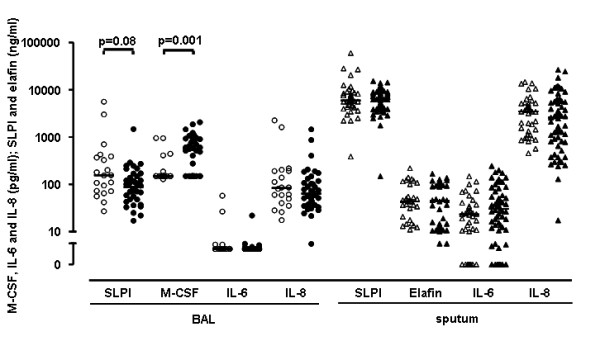
**Soluble mediators measured in BAL and induced sputum supernatants of COPD patients**. Ex-smokers are represented by open symbols and smokers by closed symbols. Horizontal bars represent medians. P-values are corrected for recovery of BAL fluid using multiple linear regression.

BAL M-CSF levels were lower in ex-smokers than current smokers (p = 0.001) (figure 5 and table 2). This difference was neither explained by differences in BAL recovery between both groups, nor by the ratio of M-CSF to anti-inflammatory macrophages. No correlation was found between recovery and BAL M-CSF levels. The anti-inflammatory mediator SLPI in BAL was inversely correlated with recovery. The pro-inflammatory mediators IL-6 and IL-8 in BAL were comparable between smokers and ex-smokers and were independent of recovery. No difference was found in induced sputum for the pro-inflammatory IL-6, IL-8 levels and the anti-inflammatory mediator elafin. The levels of SLPI, IL-6 and IL-8 in sputum were higher than the levels in BAL (all p < 0.001). M-CSF was below the lower limits of detection in induced sputum and elafin was undetectable in BAL.

**Table 2:** Soluble mediators measured in BAL and induced sputum supernatants of smokers and ex-smokers with COPD

Soluble mediator	Ex-smokers	Smokers
BAL		
SLPI (ng/ml)	156 (72-386)	87 (48-154)
M-CSF (pg/ml)	150 (150-159)	571 (150-927)
IL-6 (pg/ml)	6 (6-6)	6 (6-6)
IL-8 (pg/ml)	83 (43- 193)	64 (37-122)
Sputum		
SLPI (ng/ml)	5897 (4406-8628)	6643 (4321-8862)
Elafin (ng/ml)	44 (20-102)	44 (12-101)
IL-6 (pg/ml)	23 (10-36)	30 (11-58)
IL-8 (pg/ml)	3454 (1178-5212)	2571 (805-5900)

Data are presented as medians (IQR 25-75^th ^percentile).

### Correlation between cells, mediators and lung function

The number of CD163^+ ^macrophages in BAL correlated with FEV_1 _post-bronchodilator (%predicted) (Rs = 0.255; p = 0.05) and FEV_1_/IVC% (Rs = 0.374; p = 0.004). No correlations were found between the number and percentage CD163^+ ^macrophages in BAL and sputum and the number of packyears or the duration of smoking cessation. No correlations were found between the number of packyears or duration of smoking cessation and concentrations of all soluble mediators in BAL and induced sputum.

BAL M-CSF correlated with the number of CD163^+ ^macrophages in BAL (Rs = 0.379; p = 0.003). BAL SLPI was negatively correlated with the number and percentage of macrophages and positively correlated with the number and percentage of neutrophils in BAL (all p < 0.05). BAL SLPI and the number of CD163^+ ^macrophages correlated inversely (Rs = -0.353; p = 0.008). Sputum SLPI correlated with the number and percentage of CD163^+ ^macrophages in sputum (Rs = 0.377; p < 0.001 and Rs = 0.236; p = 0.021, respectively). Both BAL and sputum IL-8 correlated inversely with percentage macrophages, but positively with the percentage and number of neutrophils (all p < 0.05). This relation was not seen for IL-6. A trend was seen for a correlation between sputum IL-8 and the percentage of CD163^+ ^macrophages (Rs = -0.189; p = 0.061). The percentage, but not the number, of CD163^+ ^macrophages in BAL showed a trend for correlation with sputum (Rs = 0.267, p = 0.053).

## Discussion

This study is the first to show that the percentage of macrophages with anti-inflammatory, M2-type characteristics (as shown by CD163 expression) is significantly higher in BAL from ex-smokers than in current smokers with COPD. In addition, the percentage of anti-inflammatory macrophages was higher in BAL than in induced sputum, indicating a predominance of this macrophage phenotype in the periphery of the lung. BAL M-CSF correlated with the number of CD163^+ ^macrophages in BAL. The results together are in line with the hypothesis that smoking cessation causes a shift in the phenotype of luminal macrophages towards a more anti-inflammatory phenotype, which is restricted to the periphery of the lung. Although we did observe a higher percentage of M2-type macrophages in BAL from ex-smokers, this was not accompanied by a decrease in inflammatory parameters such as neutrophils and pro-inflammatory mediators.

Our study shows that ex-smokers with COPD have a higher percentage of anti-inflammatory macrophages in BAL than current smokers. Our findings on pulmonary macrophage polarization further extend previous observations. First, we discovered that macrophages recovered from induced sputum have less anti-inflammatory features than from BAL. A previous study showed that induced sputum of COPD patients contains a majority of pro-inflammatory macrophages, based on their HLA-DR expression and capacity to produce TNFα, in contrast to control subjects [30]. However, these authors only analyzed markers of pro-inflammatory macrophages and most patients used corticosteroids which may have affected the macrophage phenotype [17]. Second, we showed that ex-smokers have more anti-inflammatory macrophages in BAL than current smokers. This is in line with a recent paper, showing that never smokers compared to current smokers had higher BAL levels of CCL18, a chemokine expressed by alternatively activated macrophages [31]. Furthermore, previous studies have shown that anti-inflammatory macrophages have a higher phagocytic capacity [8,10]. Therefore our findings are in line with another study demonstrating that alveolar macrophages of current smokers with COPD show reduced phagocytosis compared to ex-smokers [19]. In addition, active smoking, but also the presence of COPD itself, may be associated with an impaired phagocytic capacity of alveolar macrophages (and therefore a predominance of pro-inflammatory macrophages) [32-34]. However, in contrast to these and our findings, a recent study indicated that smoking may enhance macrophage differentiation into an anti-inflammatory phenotype, since cigarette smoking polarized human alveolar macrophages of COPD patients *in vivo *towards an enhanced expression of M2-related genes and a suppression of M1 genes [35]. This study included only 12 COPD patients with predominantly GOLD stage I. A possible explanation for this apparent difference with our observations is therefore that the direction of the effect of smoking on macrophage differentiation may be determined by disease severity.

Previously, several studies have evaluated the effect of smoking on soluble mediators. We found comparable SLPI levels in BAL between current smokers and ex-smokers with COPD, in line with results from a study of 25 smoking, ex-smoking and never smoking COPD patients with GOLD stage II-III [36]. We did not find a difference in BAL IL-6 and IL-8 and sputum IL-6 between current smokers and ex-smokers with COPD, in line with two previous studies [37,38].

We believe that our study has several strengths. We studied a large cohort of well-characterized COPD patients in which sputum (n = 114) and BAL (n = 71) were collected, whereas previous studies were of smaller size [30,31,36,39]. In addition, we studied steroid-naive patients, excluding possible influences of inhaled corticosteroid therapy on CD163 expression. This is important, since it has been shown in previous studies that dexamethasone induces CD163 expression on monocytes and macrophages *in vitro *[17]. The BAL and sputum cytospins were counted manually by two independent researchers simultaneously (LIK and SVW). CD163^- ^macrophages as well as CD163^+ ^macrophages were readily recognized. Repeatability between the observers was good, as measured by the intraclass correlation coefficient (data not shown).

A number of limitations needs to be taken into account when interpreting our results. First, this was a cross-sectional study and it cannot be ruled out that our group of ex-smokers quit smoking because they experienced more smoking related symptoms and they may have had different macrophage phenotypes before quitting. In addition, we did not confirm smoking status by laboratory tests which is in line with other cross-sectional studies [4,5] and therefore cannot exclude the possibility that some ex-smokers were still smoking. Second, BAL samples were not available from all subjects in our study due to ethical considerations. As this was not anticipated, it is unlikely that a selection bias for the BAL results was introduced. Nevertheless, a significant difference in anti-inflammatory macrophages in BAL was found between smokers and ex-smokers. Further studies are needed to investigate whether the observed differences in CD163 staining on macrophages are also observed when comparing current or ex-smokers without COPD to non-smokers and whether CD163 expression is a specific feature of COPD. Third, we only focused on the marker CD163 for M2 macrophages, which can result in an oversimplification of our conclusions. Furthermore, it appears that the M2 macrophage population is more heterogeneous than the M1 population [9] and M2 subpopulations were not taken into account in our analysis. Obviously, it is of interest to evaluate whether the use of pro-inflammatory or other anti-inflammatory markers (like arginase or iNOS) can confirm our results and whether associated functional differences can be detected. Currently, there is no general agreement on well defined markers for M1 macrophages.

Fourth, we found that the percentage CD163^+ ^cells is higher in ex-smokers with COPD whereas the number of CD163^+ ^cells is higher in current smokers with COPD. In addition, we observed a higher percentage and number of macrophages in BAL from smokers compared to ex-smokers, which likely results from more active recruitment of monocytes from the circulation. Therefore, it is not surprising that smokers have a higher number of CD163^+ ^cells in BAL, since they have more macrophages in BAL. We hypothesize that percentages and numbers provide different and complimentary information: percentages better reflect the environment during differentiation, whereas cell numbers result from both recruitment and differentiation. Fifth, several soluble mediators were below the lower limits of detection in sputum and BAL supernatants. Finally, analysis of cytospins using immunocytochemistry is a semi-quantitative measurement and could therefore result in incorrect interpretations. Using e.g. FACS analysis ideally combined with functional analysis of e.g. the phagocytic capacity of the macrophages, could have been more accurate to evaluate the equilibrium between pro- and anti-inflammatory macrophages in our samples. Unfortunately, fresh samples were not available at the time of this research.

How can we explain our results? Macrophages in the periphery of the lung in healthy individuals display mainly anti-inflammatory characteristics that may be involved in suppressing inflammation in this area of the lung. Our study, as well as recent data from others [19,40], suggest that the anti-inflammatory environment may change into a pro-inflammatory environment as COPD develops in smokers. This is in line with the observation that IL-10 levels are lower and GM-CSF and Matrix Metalloproteinase (MMP)-12 levels are higher in sputum and BAL from COPD patients compared to healthy controls [39,41,42]. Inflammatory lung diseases, including COPD [43], are characterized by increased local production of GM-CSF which may contribute to development of a pro-inflammatory macrophage phenotype in addition to its established effect on neutrophil survival [44]. Macrophages maintain their plasticity even when differentiated into M1 or M2 cells and can switch their phenotype dependent on the presence of appropriate stimuli [45,46]. In this study we add to the field that smoking cessation may skew alveolar macrophage heterogeneity towards a more anti-inflammatory phenotype as characterized by the M2 marker CD163. Pro-inflammatory macrophages are the predominant phenotype in the central airways, which may be explained by high exposure to pathogens and environmental stimuli compared to macrophages in the peripheral airways. The higher percentage and number of neutrophils in sputum samples are in line with this observation. The predominance of anti-inflammatory macrophages in the periphery of the lung may help to keep this area, which is central to gas exchange, free from excessive inflammation.

Our results suggest that smoking cessation can change macrophage polarization from a pro-inflammatory towards a CD163 expressing anti-inflammatory phenotype, which may decrease inflammation and enhance repair. Our findings of a positive association between a better lung function and more anti-inflammatory M2 macrophages are in line with this. We hypothesize that a shift in macrophage phenotype contributes to further clinical effects of smoking cessation. Therefore, the plasticity of the macrophage phenotype and the possibility to modulate this phenotype may be relevant to the treatment of chronic inflammation, including COPD.

## Conclusions

This study shows that previous smoking cessation may contribute to the anti-inflammatory phenotype of intraluminal macrophages in BAL of ex-smoking COPD patients *in vivo*. Additional research is needed to further characterize this phenotype and to demonstrate its impact on local inflammation. Furthermore, studies are needed to investigate whether it is restricted to luminal macrophages or is also present in lung tissue. Prospective studies are required to show whether anti-inflammatory treatment contributes to the anti-inflammatory macrophage phenotype *in vivo*, and whether this contributes to treatment effects on inflammation and clinical outcomes such as lung function decline.

## Competing interests

This study was funded by Netherlands Organization for Scientific Research (NWO), Dutch Asthma Foundation (NAF), Stichting Astma Bestrijding (SAB), GlaxoSmithKline (GSK) of the Netherlands, University Medical Center Groningen (UMCG), and Leiden University Medical Center (LUMC).

## Authors' contributions

LIK, TSL, JBS, WT, PJS, DSP and PSH designed the study design and the experiments. DSP and KFR performed the bronchoscopies. JAS, SVW and LIK were responsible for immunocytochemical stainings and cell counting. LIK statistically analyzed the data. LIK and PSH drafted the manuscript. SEB, WT, PJS, KFR and DSP read, critically revised and all authors approved the final manuscript.

## References

[B1] Rabe KF Hurd S Anzueto A Barnes PJ Buist SA Calverley P Global strategy for the diagnosis, management, and prevention of chronic obstructive pulmonary disease: GOLD executive summary Am J Respir Crit Care Med 2007 176 532 555 10.1164/rccm.200703-456SO 17507545

[B2] Hogg JC Chu F Utokaparch S Woods R Elliott WM Buzatu L The nature of small-airway obstruction in chronic obstructive pulmonary disease N Engl J Med 2004 350 2645 2653 10.1056/NEJMoa032158 15215480

[B3] Barnes PJ Immunology of asthma and chronic obstructive pulmonary disease Nat Rev Immunol 2008 8 183 192 10.1038/nri2254 18274560

[B4] Rutgers SR Postma DS ten Hacken NH Kauffman HF van der Mark TW Koeter GH Ongoing airway inflammation in patients with COPD who do not currently smoke Thorax 2000 55 12 18 10.1136/thorax.55.1.12 10843943

[B5] Turato G Di Stefano A Maestrelli P Mapp CE Ruggieri MP Roggeri A Effect of smoking cessation on airway inflammation in chronic bronchitis Am J Respir Crit Care Med 1995 152 1262 1267 7551380 10.1164/ajrccm.152.4.7551380

[B6] Willemse BWM ten Hacken NHT Rutgers B Lesman-Leegte IGAT Postma DS Timens W Effect of 1-year smoking cessation on airway inflammation in COPD and asymptomatic smokers Eur Respir J 2005 26 835 845 10.1183/09031936.05.00108904 16264044

[B7] Lapperre TS Postma DS Gosman MME Snoeck-Stroband JB ten Hacken NHT Hiemstra PS Relation between duration of smoking cessation and bronchial inflammation in COPD Thorax 2006 61 115 121 10.1136/thx.2005.040519 16055612 PMC2104584

[B8] Martinez FO Helming L Gordon S Alternative activation of macrophages: an immunologic functional perspective Annu Rev Immunol 2009 27 451 483 10.1146/annurev.immunol.021908.132532 19105661

[B9] Mosser DM Edwards JP Exploring the full spectrum of macrophage activation Nat Rev Immunol 2008 8 958 969 10.1038/nri2448 19029990 PMC2724991

[B10] Gordon S Taylor PR Monocyte and macrophage heterogeneity Nat Rev Immunol 2005 5 953 964 10.1038/nri1733 16322748

[B11] Verreck FAW de Boer T Langenberg DML van der Zanden L Ottenhoff THM Phenotypic and functional profiling of human proinflammatory type-1 and anti-inflammatory type-2 macrophages in response to microbial antigens and IFN-{gamma}- and CD40L-mediated costimulation J Leukoc Biol 2006 79 285 293 10.1189/jlb.0105015 16330536

[B12] Savage NDL de Boer T Walburg KV Joosten SA van Meijgaarden K Geluk A Human anti-inflammatory macrophages induce Foxp3+GITR+CD25+ regulatory T cells, which suppress via membrane-bound TGF{beta}-1 J Immunol 2008 181 2220 2226 18641362 10.4049/jimmunol.181.3.2220

[B13] Xu W Roos A Schlagwein N Woltman AM Daha MR van Kooten C IL-10-producing macrophages preferentially clear early apoptotic cells Blood 2006 107 4930 4937 10.1182/blood-2005-10-4144 16497970

[B14] Blumenthal RL Campbell DE Hwang P DeKruyff RH Frankel LR Umetsu DT Human alveolar macrophages induce functional inactivation in antigen-specific CD4 T cells J Allergy Clin Immunol 2001 107 258 264 10.1067/mai.2001.112845 11174191

[B15] Thepen T van Rooijen N Kraal G Alveolar macrophage elimination in vivo is associated with an increase in pulmonary immune response in mice J Exp Med 1989 170 499 509 10.1084/jem.170.2.499 2526847 PMC2189410

[B16] Van den Heuvel MM Tensen CP van As JH van den Berg TK Fluitsma DM Dijkstra CD Regulation of CD 163 on human macrophages: cross-linking of CD163 induces signaling and activation J Leukoc Biol 1999 66 858 866 10577520 10.1002/jlb.66.5.858

[B17] Högger P Dreier J Droste A Buck F Sorg C Identification of the integral membrane protein RM3/1 on human monocytes as a glucocorticoid-inducible member of the scavenger receptor cysteine-rich family (CD163) J Immunol 1998 161 1883 1890 9712057

[B18] Schonkeren D van der Hoorn ML Khedoe P Swings G van Beelen E Claas FHJ Differential distribution and phenotype of decidual macrophages in preecclamptic versus control pregnancies Am J Pathol 2010 178 709 717 10.1016/j.ajpath.2010.10.011 PMC3069820 21281803

[B19] Hodge S Hodge G Ahern J Jersmann H Holmes M Reynolds PN Smoking alters alveolar macrophage recognition and phagocytic ability: Implications in chronic obstructive pulmonary disease Am J Respir Cell Mol Biol 2007 37 748 755 10.1165/rcmb.2007-0025OC 17630319

[B20] Sica A Schioppa T Mantovani A Allavena P Tumour-associated macrophages are a distinct M2 polarised population promoting tumour progression: Potential targets of anti-cancer therapy Eur J Cancer 2006 42 717 727 10.1016/j.ejca.2006.01.003 16520032

[B21] Woollard KJ Geissmann F Monocytes in atherosclerosis: subsets and functions Nat Rev Cardiol 2010 7 77 86 10.1038/nrcardio.2009.228 20065951 PMC2813241

[B22] Ricardo SD van Goor H Eddy AA Macrophage diversity in renal injury and repair J Clin Invest 2008 118 3522 3530 10.1172/JCI36150 18982158 PMC2575702

[B23] Lapperre TS Snoeck-Stroband JB Gosman MME Stolk J Sont JK Jansen DF Dissociation of lung function and airway inflammation in chronic obstructive pulmonary disease Am J Respir Crit Care Med 2004 170 499 504 10.1164/rccm.200401-112OC 15172889

[B24] Lapperre TS Willems LNA Timens W Rabe KF Hiemstra PS Postma DS Small Airways Dysfunction and Neutrophilic Inflammation in Bronchial Biopsies and BAL in COPD Chest 2007 131 53 59 10.1378/chest.06-0796 17218556

[B25] Quanjer PH Tammeling GJ Cotes JE Pedersen OF Peslin R Yernault JC Lung volumes and forced ventilatory flows. Report Working Party Standardization of Lung Function Tests, European Community for Steel and Coal. Official Statement of the European Respiratory Society Eur Respir J Suppl 1993 16 5 40 8499054

[B26] Technical recommendations and guidelines for bronchoalveolar lavage (BAL). Report of the European Society of Pneumology Task Group Eur Respir J 1989 2 561 585 2663535

[B27] Haslam PL Baughman RP Report of ERS Task Force: guidelines for measurement of acellular components and standardization of BAL Eur Respir J 1999 14 245 248 10.1034/j.1399-3003.1999.14b01.x 10515395

[B28] in't Veen JC de Gouw HW Smits HH Sont JK Hiemstra PS Sterk PJ Repeatability of cellular and soluble markers of inflammation in induced sputum from patients with asthma Eur Respir J 1996 9 2441 2447 8980951 10.1183/09031936.96.09122441

[B29] van Wetering S van der Linden AC van Sterkenburg MA Rabe KF Schalkwijk J Hiemstra PS Regulation of secretory leukocyte proteinase inhibitor (SLPI) production by human bronchial epithelial cells: increase of cell-associated SLPI by neutrophil elastase J Investig Med 2000 48 359 366 10979241

[B30] Frankenberger M Menzel M Betz R Kaßner G Weber N Kohlhäufl M Characterization of a population of small macrophages in induced sputum of patients with chronic obstructive pulmonary disease and healthy volunteers Clinical & Experimental Immunology 2004 138 507 516 10.1111/j.1365-2249.2004.02637.x PMC1809248 15544629

[B31] Kollert F Probst C Muller-Quernheim J Zissel G Prasse A CCL18 production is decreased in alveolar macrophages from cigarette smokers Inflammation 2009 32 163 168 10.1007/s10753-009-9115-5 19357939

[B32] Berenson CS Garlipp MA Grove LJ Maloney J Sethi S Impaired phagocytosis of nontypeable Haemophilus influenzae by human alveolar macrophages in chronic obstructive pulmonary disease J Infect Dis 2006 194 1375 1384 10.1086/508428 17054066

[B33] Taylor AE Finney-Hayward TK Quint JK Thomas CMR Tudhope SJ Wedzicha JA Defective macrophage phagocytosis of bacteria in COPD Eur Respir J 2010 35 1039 1047 10.1183/09031936.00036709 19897561

[B34] Verreck FAW de Boer T Langenberg DML Hoeve MA Kramer M Vaisberg E Human IL-23-producing type 1 macrophages promote but IL-10-producing type 2 macrophages subvert immunity to (myco)bacteria Proc Natl Acad Sci USA 2004 101 4560 4565 10.1073/pnas.0400983101 15070757 PMC384786

[B35] Shaykhiev R Krause A Salit J Strulovici-Barel Y Harvey BG O'Connor TP Smoking-dependent reprogramming of alveolar macrophage polarization: Implication for pathogenesis of chronic obstructive pulmonary disease J Immunol 2009 183 2867 2883 10.4049/jimmunol.0900473 19635926 PMC2873685

[B36] Hollander C Sitkauskiene B Sakalauskas R Westin U Janciauskiene SM Serum and bronchial lavage fluid concentrations of IL-8, SLPI, sCD14 and sICAM-1 in patients with COPD and asthma Respir Med 2007 101 1947 1953 10.1016/j.rmed.2007.04.010 17574828

[B37] Aaron S Vandemheen K Ramsay T Zhang C Avnur Z Nikolcheva T Multi analyte profiling and variability of inflammatory markers in blood and induced sputum in patients with stable COPD Respiratory Research 2010 11 41 10.1186/1465-9921-11-41 20412595 PMC2874769

[B38] Stravinskaite K Sitkauskiene B Dicpinigaitis PV Babusyte A Sakalauskas R Influence of smoking status on cough reflex sensitivity in subjects with COPD Lung 2009 187 37 42 10.1007/s00408-008-9124-4 18949517

[B39] Saha S Doe C Mistry V Siddiqui S Parker D Sleeman M Granulocyte-macrophage colony-stimulating factor expression in induced sputum and bronchial mucosa in asthma and COPD Thorax 2009 64 671 676 10.1136/thx.2008.108290 19213775 PMC2712140

[B40] Hodge S Matthews G Mukaro V Ahern J Shivam A Hodge G Cigarette Smoke-induced Changes to Alveolar Macrophage Phenotype and Function is Improved by Treatment with Procysteine Am J Respir Cell Mol Biol 2010 2009-0459OC 10.1165/rcmb.2009-0459OC 20595463

[B41] Takanashi S Hasegawa Y Kanehira Y Yamamoto K Fujimoto K Satoh K Interleukin-10 level in sputum is reduced in bronchial asthma, COPD and in smokers Eur Respir J 1999 14 309 314 10.1183/09031936.99.14230999 10515406

[B42] Babusyte A Stravinskaite K Jeroch J Lotvall J Sakalauskas R Sitkauskiene B Patterns of airway inflammation and MMP-12 expression in smokers and ex-smokers with COPD Respir Res 2007 8 81 10.1186/1465-9921-8-81 18001475 PMC2200652

[B43] Culpitt SV Rogers DF Shah P De Matos C Russell REK Donnelly LE Impaired inhibition by dexamethasone of cytokine release by alveolar macrophages from patients with chronic obstructive pulmonary disease Am J Respir Crit Care Med 2003 167 24 31 10.1164/rccm.200204-298OC 12406856

[B44] Lee E Lindo T Jackson N Meng-Choong L Reynolds P Hill A Reversal of human neutrophil survival by leukotriene B4 receptor blockade and 5-lipoxygenase and 5-lipoxygenase activating protein inhibitors Am J Respir Crit Care Med 1999 160 2079 2085 10588632 10.1164/ajrccm.160.6.9903136

[B45] Yanagita M Kobayashi R Murakami S Nicotine can skew the characterization of the macrophage type-1 (MPhi1) phenotype differentiated with granulocyte-macrophage colony-stimulating factor to the MPhi2 phenotype Biochem Biophys Res Commun 2009 388 91 95 10.1016/j.bbrc.2009.07.124 19646418

[B46] Stout RD Jiang C Matta B Tietzel I Watkins SK Suttles J Macrophages sequentially change their functional phenotype in response to changes in microenvironmental influences J Immunol 2005 175 342 349 15972667 10.4049/jimmunol.175.1.342

